# Zenker's Diverticulum: Carbon Dioxide Laser Endoscopic Surgery

**DOI:** 10.1155/2014/516231

**Published:** 2014-03-06

**Authors:** Jan Plzák, Michal Zábrodský, Petr Lukeš

**Affiliations:** Department of Otorhinolaryngology and Head and Neck Surgery, 1st Faculty of Medicine, Charles University, University Hospital Motol, V Úvalu 84, 150 06 Prague 5, Czech Republic

## Abstract

Nowadays endoscopic diverticulotomy is the surgical approach of the first choice in treatment of Zenker's diverticulum. We report our experience with this procedure and try to sum up recent recommendations for management of surgery and postoperative care. Data of 34 patients with Zenker's diverticulum, treated by endoscopic carbon dioxide laser diverticulotomy at the Department of Otorhinolaryngology and Head and Neck Surgery, 1st Faculty of Medicine, Charles University, University Hospital Motol, Prague, Czech Republic, were prospectively stored and followed in relatively short period from May 2009 to December 2013. The average length of diverticulum was 32 mm. The average duration of surgery was 32 min. The patients were fed via feeding tube for 6.1 days and antibiotics were administered for 7 days. Mean hospitalization time was 7.4 days. We observed one transient recurrent laryngeal nerve paralysis and no other serious complications. Recurrence rate was 3%. We recommend complete transection of the diverticular septum in one procedure, systemic antibiotic treatment and exclusion of transoral intake for minimally 5 days, and contrast oesophagogram before resumption of oral intake to exclude fistula. Open diverticulectomy should be reserved for cases with inadequate endoscopic exposure and for revision surgery for multiple recurrences from endoscopic diverticulotomies.

## 1. Introduction

Hypopharyngeal diverticulum (Zenker's diverticulum) is a herniation of the posterior wall of the hypopharynx into a triangular shaped area between the oblique muscle fibers of the inferior pharyngeal constrictor muscle and the horizontal muscle fibers of the cricopharyngeal muscle. Although the exact etiology remains unclear, mostly accepted explanation of hypopharyngeal diverticulum development is an increased bolus pressure as a result of a malfunction of the upper oesophageal sphincter, including spasms, lack of relaxation during swallowing, or premature contraction.

Symptoms, related to the size of the diverticulum, range from complete absence of any complaints to life-threatening situation such as aspiration pneumonia and severe cachexia. Dysphagia is the main presenting symptom, sometimes associated with regurgitation of undigested food, choking, cough, and, in advanced cases, oesophageal obstruction. The diagnosis is confirmed by radiological examination.

The treatment of choice for Zenker's diverticulum is surgery. External diverticulectomy with cricopharyngeal myotomy, firstly performed by Kaplan in 1951 [1], is the key point of modern surgical management. To reduce rate of complications (e.g., mediastinitis, recurrent laryngeal nerve paralysis, and pharyngocutaneous fistulas) and to shorten hospitalization time, endoscopic approaches have been developed. The great expansion of endoscopic treatment started after introduction of carbon dioxide laser in 1981 by van Overbeek [2]. The great leap forward has been supported by endoscopic stapler-assisted diverticulotomy, introduced more recently in 1993 separately by Martin-Hirsch and Newbegin and Collard et al. [3, 4]. Nowadays endoscopic diverticulotomy is the surgical approach of the first choice in treatment of Zenker's diverticulum [5].

Up to now, a number of studies regarding both external and endoscopic approaches have been published, showing some differences in management and results such as complication rate, duration of hospitalization, exact treatment protocol, and followup. In this study, we present our experience with carbon dioxide laser endoscopic diverticulotomy in 34 patients in less than five-year period.

## 2. Material and Methods

Data of 34 patients with Zenker's diverticulum treated by endoscopic carbon dioxide laser diverticulotomy at the Department of Otorhinolaryngology and Head and Neck Surgery, 1st Faculty of Medicine, Charles University, University Hospital Motol, Prague, Czech Republic, were prospectively stored and followed from May 2009 to December 2013.The study was conducted with patient consent and approval of the Local Ethical Committee according to the principles of the Helsinki Declaration. Data were analyzed for age, sex, size of the diverticulum, duration of surgery, length of feeding tube nutrition, length of hospitalization stay, and complications.

Diagnosis was based on the patient's history, complete clinic otorhinolaryngological examination, and contrast oesophagogram. The size of diverticulum was defined by the maximal depth of the diverticular sac on the preoperative contrast oesophagogram. Duration of surgery was recorded as the time from when the surgeon initiates the procedure till the completion of the procedure. All patients with diagnosed Zenker's diverticulum were in this period indicated to endoscopic surgery except three ones, one patient with huge diverticulum of 8 cm length extended deep in the upper mediastinum; the second one because of patient's preference; the third one as a conversion from endoscopic approach due to unfavourable anatomy.

The surgery was performed under general anesthesia. Initially classic rigid oesophagoscopy was performed to prove typical location of diverticular inlet at the posterior wall of the hypopharynx, to clean the diverticular sac from any food debris, and to exclude cancer. Subsequently a Weerda distending diverticuloscope (Karl Storz, Tuttlingen, Germany) was used for diverticulum exposure. The anterior lip of the diverticuloscope was placed into the oesophagus while the posterior lip of the diverticuloscope was passed into the diverticulum. The diverticuloscope was advanced until the bottom of the diverticulum was exposed. The tissue bridge between the oesophagus anteriorly and the diverticulum posteriorly was set between the two lips of the diverticuloscope. An operating microscope Carl Zeiss OPMI Sensera (Carl Zeiss AG, Oberkochen, Germany) with attached carbon dioxide laser micromanipulator was set on working distance 400 mm with the laser beam focused on the tissue bridge. We used a carbon dioxide laser Lumenis AcuPulse (Lumenis, Santa Clara, California) with super-pulse delivery in repeated mode, coupled to an AcuSpot micromanipulator, until 2009. Since March 2010, a robotic digital AcuBlade scanning micromanipulator system was used. The oesophageal mucosa was protected from accidental laser injury by a moist swab. Using the laser at 5–10 W, the septum was transected at the midline down to the bottom of diverticular sac. Occasionally electrocautery was used to control bleeding. Feeding tube was introduced in all patients.

Postoperative oesophagogram was performed at 5-6th day followed by a removal of the feeding tube and the discharge from the hospital at the same day or one day later. Antibiotic treatment was routinely administered for one week following the surgery (cefuroxime axetil). Control contrast oesophagogram and subjective evaluation of swallowing were performed at least three months after the treatment.

## 3. Results 

**Figure 1:**
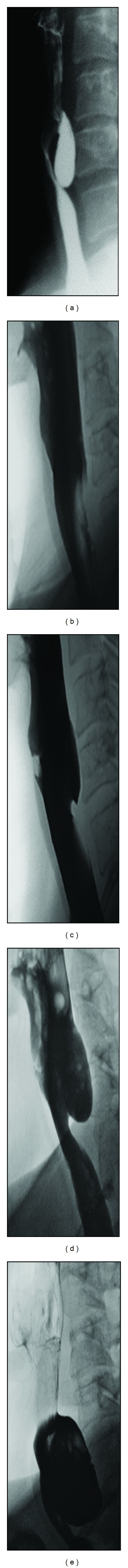
Failure case. Contrast oesophagograms before the first laser endoscopic diverticulotomy (a), 5th postoperative day (b), 3 months postoperatively (c), 6 months postoperatively (d), and 15 months postoperatively (e) show slow recurrence to the preoperative size of the Zenker's diverticulum.

The group of 34 patients with Zenker's diverticulum treated by endoscopic carbon dioxide laser diverticulotomy by three surgeons, who are the authors of this article, included 25 males and 9 females, mean age 63 years (range 36–91). One male patient required a revision endoscopic surgery 18 months after the first diverticulotomy of 28 mm sac due to a deterioration of swallowing accompanied by progressing recurrent diverticulum showed at X-ray (Figure 1). Therefore, data of 35 procedures are presented. In one female patient diverticular septum could not be exposed well due to unfavourable anatomy obesity, short neck, enlargement of the base of the tongue, and high upper teeth. The successful diverticulectomy was performed using transcervical approach three days after the endoscopic attempt. Two patients had been previously treated by external transcervical approach. One patient initially preferred external approach at our department, but the surgery was not successful with complicated course with fistula. Revision endoscopic surgery was uneventful. The second patient underwent external approach at another institution. Fistula and transient recurrent nerve paralysis was complication of this surgery with minimal release of swallowing difficulties and unchanged X-ray picture. Also in this case revision endoscopic surgery was uneventful and successful. One patient also had a resection of recurrent glottic papillomas performed by carbon dioxide laser, in addition to the diverticulotomy.

The average length of diverticulum was 32 mm (range 22–52 mm). According to the Brombat classification [6], 3 cases were classified stage III, and 31 cases stage IV. The average duration of surgery was 32 min (range 17–45 min). Mean hospitalization time was 7.4 days (range 7–14 days). All cases had hospitalization time between 7 and 9 days, except one that was admitted one week before the surgery for preoperative workup, because of complicated comorbid conditions. All but one patient were fed by a nasogastric feeding tube on average for 6.1 days (range 6-7 days). The one patient removed the feeding tube accidentally by himself during the first postoperative night and hence he was subsequently fed parenteraly for 7 days.

No case of fistula, mediastinitis, neck emphysema, mucosal perforation or tearing, tooth fracture, postoperative bleeding, and aspiration pneumonia was observed. One patient suffered from transient left recurrent laryngeal nerve paralysis that spontaneously resolved within one month. Once we observed oedema of the laryngeal inlet and left pyriform sinus that required intravenous application of corticosteroids for one day, five patients (14%) presented in 24 hours after the surgery a temperature peak > 38°C that resolved with antipyretic treatment. Elevated CRP (C-reactive protein) was observed in all cases. The average maximum CRP was 52 (range 17–224).

All contrast oesophagograms at 5-6th postoperative day showed no leakage and no presence of extraoesophageal air in the neck and mediastinum. In 25 cases (71%) it was possible to identify previous location of the pouch inlet as an evagination at the posterior pharyngooesophageal wall. Contrast oesophagogram three months after the surgery revealed radiographic recurrence of Zenker's diverticulum in 4 patients (11%). Two of them evaluated their swallowing as satisfying and improved, with size of recurrent pouch of 4 and 5 mm, respectively. They were recommended for control radiogram in case of deterioration of swallowing but no one required it. One patient described complete regression of swallowing problems immediately after the surgery with some deterioration later on. Control X-ray showed progression of recurrent diverticulum to preoperative diameter accompanied by a deterioration of dysphagia that was graded by the patient as slighter than preoperative one. The patient underwent revision endoscopic diverticulotomy. One patient suffered from persistent postoperative dysphagia but without previously presented regurgitation of undigested food, gurgling in the throat, and other typical symptoms of a Zenker's diverticulum. Moreover, the length of recurrent diverticulum was only 6 mm with horizontal course of a pouch and no retention of contrast medium in it. Revision surgery was not recommended since problems of the patient could not be solved by diverticular surgery. All other patients evaluated their swallowing function as normal or much lighter symptoms.

We assessed as a failure one patient with recurrent both dysphagia and X-ray finding that required revision surgery. Total success rate was 97% (33/34 patients) including two revision procedures after previous open surgery. Only the first (i.e., unsuccessful) endoscopic procedure of patient with failure is counted in the success rate since there is a short time from revision surgery to evaluate it correctly.

## 4. Discussion 

The treatment of choice for Zenker's diverticulum is surgery. Both the open transcervical and endoscopic approach are associated with complications and potential risks. The principle of the external approach is an excision of the pouch and a cricopharyngeal myotomy. The endoscopic approach, on the other hand, is based on a full-length mucosal incision and complete myotomy of the tissue bridge between the diverticular pouch and the oesophagus. Usually laser or stapling device is used thus providing wide opening of the diverticulum into the oesophagus, preventing any further retention of food. Nowadays endoscopic surgery is the approach of the first choice [5]. In favour of endoscopy comparing to open surgery, speaks these arguments: no external scar, lower complication rate (fistula, recurrent laryngeal nerve palsy, and oesophageal stenosis), shorter surgical time, shorter hospitalization time, and nonincreasing risk of revision surgery. Both approaches are highly effective (more than 90%), with slightly better results of open surgery [5, 7, 8].

Failure to expose sufficiently the oesophagus, diverticular septum and diverticulum may preclude safe transection of the septum between the oesophagus and diverticulum. In such case, conversion to transcervical procedure is indicated. Short necks, decreased hyomental distance, and/or a high BMI are anatomic factors proven to be associated with failed endoscopic exposure of Zenker's diverticulum. An open approach should be considered in this patient population [9]. The introduction of diverticuloscope in correct position enabling sufficient and safe exposure of surgical field is often, in fact, the most difficult and time consuming part of the procedure.

In our patient series, we used radiological swallowing examination in all patients 5-6th postoperative day and three months postoperatively. The first examination is aimed to exclude a fistula before resumption of oral intake. In compliance with Helmstaedter et al. we prefer to feed patients via feeding tube for around 6 days, as scar and granulation tissue need five days to seal the cut edges [10]. The second radiological examination three months after the surgery depicts final functional results. But it was shown by Mantsopoulos et al. that postoperative oesophagogram cannot predict subjective symptoms [11]. They found only 12.5% patients with pouch remnants on postoperative oesophagogram developing symptomatic recurrent Zenker's diverticulum. Therefore, for evaluation of success rate of the surgery the most important factor is subjective assessment of swallowing function done by the patient. Both primary and revision indications for surgery are guided by the patient's symptoms [12]. Based on these findings supported by our own present experience, we intend to reserve delayed postoperative oesophagogram for symptomatic cases only in future to avoid unnecessary examination.

Postoperative fever and elevation of CRP were frequent in our study (14 and 100%, resp.). Even in one case CRP peak reached 224 at 2nd postoperative day. But there were no other alerting symptoms or findings (no fewer, no chill, no thoracic pain, etc.) so we continued in standard postoperative care and CRP decreased below 20 at the date of discharge. It is considered to be due to mediastinal irritation rather than mediastinal infection. In presence of other subjective symptoms of mediastinitis like increasing chest pain, chills, general discomfort, and shortness of breath we recommend to perform thoracic CT to exclude mediastinitis.

The reason of temporary recurrent nerve palsy after endoscopic diverticulotomy is not clearly explained. We suggest that it is because of compression by the diverticuloscope and/or endotracheal tube.

Our failure case had no parameter appearing to discriminate this case from those with a good outcome. It is interesting to follow four postoperative oesophagograms. The first one showed ideal situation on the 5th day after endoscopic diverticulotomy with no residual sac. The examinations 3 and 6 months postoperatively showed recurrent pouch of 3 and 8 mm, respectively, accompanied by recurrence of dysphagia. The forth oesophagogram 15 months postoperatively reveals almost the same picture as before primary surgery with typical Zenker's diverticulum of size equal to preoperative one accompanied by further deterioration of swallowing function (Figure 1). But the patient assessed dysphagia before primary surgery as more serious than before revision surgery. This case is in accordance with a statistical analysis of a huge group of 155 cases made by Mantsopoulos et al. showing that early postoperative oesophagogram does not have any prognostic value [11]. They concluded that the presence of a residual diverticulum on immediate postoperative oesophagogram does not justify early surgical revision, even when dysphagia persists. In many cases, there may be a slow process of gradual fibrosis of the residual dysfunctional cricopharyngeal muscle or atrophy of the pouch remnant over the course of time. Our case may support their result from the other point of view: even normal postoperative oesophagogram probably cannot predict a persistent good outcome.

So far published recurrence rate of endoscopic laser diverticulotomy is around 5–25% [5, 8, 12–14]. Our recurrence rate is 3%. This low number may be explained by smaller group of patients and short followup. On the other hand we found only one report with higher rate of endoscopic laser diverticulotomies per year than ours. Adam et al. performed 148 surgeries in 10 years (14.8/year), Koch et al. performed 101 surgeries in 18 years (5.6/year), Rizzetto et al. performed 51 surgeries in 14 years (3.6/year), Verhaegen et al. performed 72 surgeries in 20 years (3.6/year), Helmstaedter et al. performed 40 surgeries in 20 years (2/year), and Peretti et al. performed 28 surgeries in 15 years (1.9/year) [8, 10, 12–15]. We performed 35 surgeries in 4.67 years (7.5/year). This relatively high frequency of endoscopic laser diverticulotomy per year is in favour of us since the positive correlation of high frequency and high efficacy/low complication rate of any surgery due to increased familiarity is well known. Our other advantage is that we report a group of patients from very recent period, that is, after 2009. Thus we may utilize up-to-date knowledge. In the past some authors advocated transecting the diverticular septum for only 2–4 cm irrespective of the size of the diverticulum to avoid risk of opening of the mediastinum and subsequent mediastinitis. [16, 17]. But it has been already demonstrated that complete transection of the diverticular septum does not increase the risk of complication [5, 10]. Therefore, we performed complete transection in all cases irrespective of the size of diverticulum.

In accordance with the recent literature we recommend endoscopic procedure also for revision surgery. All our three revision cases (two cases after primary external surgery with complications, one after our primary endoscopic surgery that is discussed in more detail; see above) passed uneventfully, with standard course of surgery and no complications. The great advantage compared to external approach is that endoscopic revision treatment is technically feasible and relatively easy, no more difficult than the primary endoscopic procedure, with no increase of risk. Moreover, patient satisfaction was higher in those who underwent endoscopic revision surgery compared to those who underwent open revision surgery [5, 12, 18].

## 5. Conclusions

Endoscopic carbon dioxide laser diverticulotomy in consecutive series of 34 patients was safe, fast, and successful treatment of patients with Zenker's diverticulum. Recurrence rate of 3% and morbidity rate of 3% (1x transient recurrent laryngeal nerve palsy, no fistula, and no mediastinitis) support the protocol we used. We recommend maximal effort to reach complete transection of the diverticular septum in one procedure, systemic antibiotic treatment, and exclusion of transoral intake for minimally 5 days. We recommend contrast oesophagogram before resumption of oral intake to exclude fistula. Unfortunately there is currently no radiologic prognostic marker of recurrence of the disease. Open transcervical diverticulectomy should be reserved for cases with inadequate endoscopic exposure of the operation field due to unfavourable anatomy and for revision surgery for multiple recurrences from endoscopic diverticulotomies.
